# Impact of clinicopathological factors on extended endocrine therapy decision making in estrogen receptor–positive breast cancer

**DOI:** 10.3389/fonc.2022.996522

**Published:** 2023-01-12

**Authors:** Weilin Chen, Jiayi Wu, Yifei Zhu, Jiahui Huang, Xiaosong Chen, Ou Huang, Jianrong He, Yafen Li, Weiguo Chen, Kunwei Shen, Li Zhu

**Affiliations:** ^1^ Department of General Surgery, Comprehensive Breast Health Center, Ruijin Hospital, Shanghai Jiaotong University School of Medicine, Shanghai, China; ^2^ Department of Thyroid and Breast Surgery, Shanghai General Hospital, Shanghai Jiaotong University School of Medicine, Shanghai, China

**Keywords:** breast malignancy, extended endocrine therapy, multidisciplinary team, estrogen receptor positive (ER+), CTS5

## Abstract

**Purpose:**

In our study, we aim to analyze the impact of clinicopathological factors on the recommendation of extended endocrine therapy (EET) in patients with ER+ breast cancer and to retrospectively validate the value of CTS5 in EET decision making.

**Patients and methods:**

The retrospective analysis was performed in patients with ER+ breast cancer who have finished 4.5–5 years of adjuvant endocrine therapy and undergone MDT discussion from October 2017 to November 2019. Multivariate logistic regression was used to identify the independent factors for treatment recommendation. CTS5 was calculated for retrospective validation of the EET decision making.

**Results:**

Two hundred thirty-five patients were received; 4.5–5 years of adjuvant endocrine therapy were included in the study. Multivariate analysis suggested that age (OR 0.460, 95% CI 0.219–0.965, *p =* 0.04), pN (OR 39.350, 95% CI 9.831–157.341, *P* < 0.001), and receipt of chemotherapy (OR 3.478, 95% CI 1.336–9.055, *p =* 0.011) were independent predictors for the recommendation of EET. In the previously selective estrogen receptor modulator (SERM)–treated subgroup, pN and receipt of chemotherapy were independent predictors for the recommendation of EET. In the previously AI-treated subgroup, age, pN, and receipt of chemotherapy were independent predictors. Adverse events did not affect the recommendation in patients previously treated with adjuvant endocrine treatment nor in the previously SERM or AI-treated subgroups. CTS5 (OR 21.887, 95% CI 2.846–168.309, *p =* 0.003) remained an independent predictor for the recommendation of EET.

**Conclusions:**

Our study indicated that age, lymph nodal status, and receipt of chemotherapy were independent predictors for the recommendation of EET. The application of the CTS5 on EET decision making might be valuable among ER+ breast cancer patients.

## Introduction

For decades, breast cancer has been the most frequently diagnosed malignant tumor in women globally. According to the latest global epidemiological cancer survey, 2.1 million new cases of breast cancer were diagnosed worldwide in 2018 (1). In estrogen receptor (ER)–positive early breast cancer, endocrine therapy plays an important role in its comprehensive treatment, and 5 years of treatment was considered the standard treatment duration traditionally (2–4).

However, recent studies have shown that among women with ER-positive breast cancer who were scheduled to receive 5 years of endocrine therapy, distant recurrences still have a steady rate for at least another 15 years after the end of the 5-year treatment (5–7). According to the results of several clinical trials regarding extended adjuvant endocrine therapy (ATLAS, aTTom, MA-17R, and NSABP B-42), the effect of extended endocrine therapy (EET) beyond 5 years to reduce the risk of late recurrence for ER+ breast cancer has been demonstrated (8–13). An EBCTCG meta-analysis also showed the efficacy of extending AI therapy compared with stopping AI after about 5 years of endocrine therapy in preventing disease recurrence and death from breast cancer (14). In the American Society of Clinical Oncology (ASCO) clinical practice guideline in 2018, EET was included among node-positive and some node-negative breast cancer patients with co-existing high-risk factors (15). However, controversies remain about the target population who may benefit from EET in clinical decision making.

In our study, we aim to analyze the impact of clinicopathological factors on the choice of follow-up treatment after 5 years of endocrine therapy in patients with ER-positive breast cancer and to retrospectively validate the value of CTS5 in EET decision making.

## Patients and methods

### Study population

The retrospective analysis was performed in patients who met the following eligibility criteria: (1) female gender; (2) post-surgery; (3) have received adjuvant endocrine therapy for 4.5–5 years; (4) have undergone Multiple Disciplinary Team (MDT) discussion regarding the use of EET in Comprehensive Breast Health Center, Ruijin Hospital, Shanghai Jiao Tong University School of Medicine, Shanghai, China, between October 2017 and November 2019; (5) ER-positive. Patient information was extracted from Shanghai Jiao Tong University Breast Cancer Database (SJTU-BCDB).

### Histopathological evaluation

Tumor histopathologic result was independently performed by two experienced pathologists, including estrogen receptor (ER), progesterone receptor (PR), human epidermal growth factor receptor 2 (HER-2) status, Ki-67 status, histological grade, and pathological type. ER-positivity (ER+) and PR-positivity (PR+) were defined as more than 1% positive invasive tumor cells with nuclear staining (16). HER-2 status was identified according to the 2013 ASCO/CAP guidelines (17) (the minority of patients’ HER-2 status diagnosed before 2013 was evaluated according to 2007 ASCO/CAP guidelines (18)). The median Ki-67 value for hormone receptor-positive disease in SJTU-BCDB was 15.0%, so we defined Ki-67 high as more than 15% positive invasive tumor cells with nuclear staining. TNM stage was based on the 7^th^ edition of the American Joint Committee on Cancer (AJCC) (19). CTS5 was calculated for retrospective validation of the EET decision making, and patients were divided into two risk groups according to CTS5 score: low (< 3.13) and high (≥ 3.13) groups (20). (CTS5 = 0.438 × nodes + 0.988 × (0.093 × size(mm) - 0.001 × size (2) + 0.375 × grade + 0.017 × age).

### Treatment decision

After the completion of 4.5–5 years of adjuvant endocrine therapy, the MDT meeting would be held to recommend extending the endocrine treatment regimen based on patients’ clinicopathological features and other related factors such as adverse events. Treatment choices on whether or not to extend endocrine therapy were decided through MDT meetings including surgical oncologists, medical oncologists, radiation oncologists, ultrasound physicians, pathologists, breast cancer specialized nurses, and other related specialists. The recommendation was first determined by each physician in the MDT team and then finally determined after MDT discussion and comprehensive opinions. The standard regimens for recommendation include stopping endocrine therapy, treating with aromatase inhibitors (AIs) for 3 or 5 years, and treating with selective estrogen receptor modulators (SERMs) for 5 years, with or without applying ovarian function suppression (OFS).

### Statistical analysis

All clinicopathological characteristics were analyzed as categorical variables by using logistic regression. Multivariate logistic regression was used to identify the independent factors for treatment recommendation. The chi-square test was used to evaluate the adverse events. Fisher’s exact tests were carried out if necessary. Data were analyzed using IBM SPSS statistics software version 23 (SPSS, Inc. Chicago, IL). Two-sided *P* < 0.05 were considered statistically significant.

## Results

### Clinical characteristics

A total of 252 patients participated in the multidisciplinary discussion, 235 patients who had received 4.5–5 years of adjuvant endocrine therapy were included in the study, and 17 patients were excluded because of information loss. One hundred forty-three patients (60.9%) were suggested to receive EET, and 92 patients (39.1%) were suggested to stop EET. The mean age of patients was 60 years old, and 136 (57.9%) patients were older than 50 years. There were 140 (59.6%) patients with T1 stage tumors and 105 (44.7%) patients with positive lymph nodes. The proportion of patients with PR-positive, Ki-67 ≥ 15%, or HER-2 positive was 81.3, 51.1, and 18.7%, respectively. The baseline characteristics of the participants are presented in **Table 1**.

**Table 1:** Baseline characteristics of study participants and impact factors for extended endocrine therapy decision.

	Number	Percent %
Recommend	235	
No-EET	92	39.1
EET	143	60.9
Age, years		
≤ 60	99	42.1
> 60	136	57.9
Menopause status		
Pre	55	23.4
Post	180	76.6
pT		
pT1	140	59.6
pT2+	95	40.4
pN		
pN0	130	55.3
pN1+	105	44.7
PR status		
Negative	44	18.7
Positive	191	81.3
HER-2 status		
Negative	191	81.3
Positive	44	18.7
Ki67 status		
< 15	115	48.9
≥ 15	120	51.1
Grade		
N/A	37	15.7
I	24	10.2
II	129	54.9
III	45	19.1
Operation methods		
Lumpectomy	77	32.8
Mastectomy	158	67.2
Chemotherapy		
No	86	36.6
Yes	149	63.4
Target therapy		
No	199	84.7
Yes	36	15.3
Radiotherapy		
No	96	40.9
Yes	139	59.1
CTS5 (*N* = 198)		
< 3.13	156	78.8
≥ 3.13	42	21.2
RS (*N* = 21)		
Low risk	2	9.5
Intermediate risk	15	71.4
High risk	4	19

PR, progesterone receptor; CTS5, the Clinical Treatment Score post–5 years.

### Impact factors on decision making in all patients

In univariate analysis, age (OR 5.19, 95% CI 0.301–0.895, *p =* 0.018), pT (OR 3.042, 95% CI 1.713–5.404, *P* < 0.001), pN (OR 26.444, 95% CI 1.713–5.404, *p* < 0.001), HER-2 (OR 2.989, 95% CI 1.361–6.562, *p =* 0.006), Ki-67 status (OR 2.574, 95% CI 1.500–4.415, *p =* 0.001), Grade (GII *vs.* GI: OR 1.994, 95% CI 0.828–4.802, *p =* 0.0124; and GIII *vs.* GI: OR 6.416, 95% CI 2.506–20.016, *p =* 0.001), receipt of chemotherapy (OR 9.288, 95% CI 5.042–17.108, *p* < 0.001), receipt of target therapy (OR 3.089, 95% CI 1.292–7.387, *p =* 0.011), and receipt of radiotherapy (OR 2.510, 95% CI 1.463–4.307, *p =* 0.001) were correlated with the recommendation of EET (**Table 2**).

**Table 2:** Univariate analysis of impact factors for extended endocrine therapy recommendation in the whole-patients group.

*N* = 235	EET recommendation		OR	*p*	CI 95%
	No-EET	EET			
**Age, years**			**0.519**	**0.018**	**0.301–0.895**
**≤ 60**	**30 (30.3%)**	**69 (68.7%)**			
**> 60**	**62 (45.6%)**	**74 (54.4%)**			
Menopause status			0.774	0.425	0.412**–**1.453
Pre	19 (34.5%)	36 (65.5%)			
Post	73 (40.6%)	107 (59.4%)			
**pT**			**3.042**	< **0.001**	**1.713–5.404**
**pT1**	**69 (49.3%)**	**71 (50.7%)**			
**pT2+**	**23 (24.2%)**	**72 (75.8%)**			
**pN**			**26.444**	< **0.001**	**11.329–61.726**
**pN0**	**85 (65.4%)**	**45 (34.6%)**			
**PN1+**	**7 (6.7%)**	**98 (93.3%)**			
PR status			0.974	0.938	0.497**–**1.908
Negative	17 (38.6%)	27 (61.4%)			
Positive	75 (39.3%)	116 (60.7%)			
**HER-2 status**			**2.989**	**0.006**	**1.361–6.562**
**Negative**	**83 (43.5%)**	**108 (56.5%)**			
**Positive**	**9 (20.5%)**	**35 (79.5%)**			
**Ki67 status**			**2.574**	**0.001**	**1.500–4.415**
**< 15**	**58 (50.4%)**	**57 (49.6%)**			
**≥ 15**	**34 (28.3%)**	**86 (71.7%)**			
**Grade**				**0.004**	
**I**	**13 (54.2%)**	**11 (45.8%)**			
**II**	**48 (37.2%)**	**81 (62.8%)**	**1.994**	**0.124**	**0.828–4.802**
**III**	**7 (15.6%)**	**38 (84.4%)**	**6.416**	**0.001**	**2.056–20.016**
Operation methods			1.477	0.168	0.849**–**2.569
Lumpectomy	35 (45.5%)	42 (54.5%)			
Mastectomy	57 (36.1%)	101 (63.9%)			
**Chemotherapy**			**9.288**	**< 0.001**	**5.042–17.108**
**No**	**61 (70.9%)**	**25 (29.1%)**			
**Yes**	**31 (20.8%)**	**118 (79.2%)**			
**Target therapy**			**3.089**	**0.011**	**1.292–7.387**
**No**	**85 (42.7%)**	**114 (57.3%)**			
**Yes**	**7 (19.4%)**	**29 (80.6%)**			
**Radiotherapy**			**2.510**	**0.001**	**1.463–4.307**
**No**	**50 (52.1%)**	**46 (47.9%)**			
**Yes**	**42 (30.2%)**	**97 (69.8%)**			
**CTS5**			**30.865**	**0.001**	**4.140–230.102**
**< 3.13**	**67 (42.9%)**	**89 (57.1%)**			
**≥ 3.13**	**1 (2.4%)**	**41 (97.6%)**			
RS					0.622
Low risk	2 (100%)	0 (0)			
Intermediate risk	8 (53.3%)	7 (46.7%)	–	1	–
High risk	1 (25.0%)	3 (75.0%)	–	1	–

PR, progesterone receptor; CTS5, the Clinical Treatment Score post–5 years. The bold values provided for making meaningful result (p<0.05) stand out.

Multivariate analysis suggested that age (OR 0.460, 95% CI 0.219–0.965, *p =* 0.04), pN (OR 39.350, 95% CI 9.831–157.341, *p* < 0.001), and receipt of chemotherapy (OR 3.478, 95% CI 1.336–9.055, *p =* 0.011) were independent predictors for the recommendation of EET (**Table 3**).

### Impact factors of decision making in previously SERM/AI-treated subgroups

In univariate analysis of the previously SERM-treated group, pT (OR 4.000, 95% CI 1.136-14.085, *p =* 0.031), pN (OR 18.692, 95% CI 13.760–92.926, *p* < 0.001), Ki-67 index (OR 4.846, 95% CI 1.515–15.504, *p =* 0.008), receipt of chemotherapy (OR 16.333, 95% CI 4.281–62.310, *p* < 0.001) were correlated with EET (**Table 4**). Multivariate analysis suggested that pN (OR 10.811, 95% CI 1.937–60.346, *p =* 0.007) and receipt of chemotherapy (OR 9.396, 95% CI 2.155–40.980, *p =* 0.003) were independent predictors for the recommendation of EET (**Table 3**).

**Table 3:** Multivariate analysis of impact factors for extended endocrine therapy recommendation in whole-patients group and previously SERM or AI-treated subgroups.

		OR	95%CI	*p*
Whole patients	Age	0.460	0.219–0.965	0.04
	pN	20.695	8.099–52.882	<0.001
	Chemotherapy	5.652	2.696–11.850	<0.001
	CTS5	21.887	2.846–168.309	0.003
SERM	pN	10.811	1.937–60.346	0.007
	Chemotherapy	9.396	2.155–40.980	0.003
AI	Age	0.315	0.117–0.848	0.022
	pN	20.533	7.249–58.158	<0.001
	Chemotherapy	4.387	1.893–10.169	0.001
	CTS5	25.191	3.240–195.841	0.002

CTS5, the Clinical Treatment Score post–5 years.

**Table 4:** Univariate analysis of impact factors for extended endocrine therapy recommendation in SERM group.

SERM(*n* = 60)	EET recommendation		OR	*p*	CI 95%
	No-EET	EET			
Age, years			1.541	0.716	0.150–15.930
≤ 60	19 (33.9%)	27 (66.1%)			
> 60	1 (25.0%)	3 (75.0%)			
Menopause status			2.154	0.238	0.603–7.699
Pre	16 (38.1%)	26 (61.9%)			
Post	4 (22.2%)	14 (77.8%)			
**pT**			**4.000**	**0.031**	**1.136–14.085**
**pT1**	**16 (44.4%)**	**20 (55.6%)**			
**pT2+**	**4 (16.7%)**	**20 (83.3%)**			
**pN**			**18.692**	**< 0.001**	**13.760–92.929**
**pN0**	**18 (58.1%)**	**13 (41.9%)**			
**pN1+**	**2 (6.9%)**	**27 (93.1%)**			
PR status			0.778	0.777	0.137–4.412
Negative	2 (28.6%)	5 (71.4%)			
Positive	18 (34.0%)	35 (66.0%)			
HER-2 status			3.857	0.100	0.771–19.293
Negative	18 (39.1%)	28 (60.9%)			
Positive	2 (14.3%)	12 (85.7%)			
**Ki67 status**			**4.846**	**0.008**	**1.515–15.504**
**< 15**	**14 (51.9%)**	**13 (48.1%)**			
**≥ 15**	**6 (18.2%)**	**27 (81.8%)**			
**Grade**				0.374	
**I**	3 (37.5%)	5 (62.5%)			
**II**	8 (25.0%)	24 (75.0%)	1.800	0.482	0.349–9.278
**III**	1 (9.1%)	10 (90.9%)	6.000	0.161	0.490–73.452
Operation methods			1.420	0.551	0.449–4.490
Lumpectomy	7 (38.9%)	11 (61.1%)			
Mastectomy	13 (31.0%)	29 (69.0%)			
**Chemotherapy**			**16.333**	**< 0.001**	**4.281–62.310**
**No**	**14 (73.7%)**	**5 (26.3%)**			
**Yes**	**6 (14.6%)**	**35 (85.4%)**			
Target therapy			6.333	0.090	0.749–53.531
No	19 (38.8%)	30 (61.2%)			
Yes	1 (9.1%)	10 (90.9%)			
Radiotherapy			2.500	0.103	0.832–7.511
No	12 (44.4%)	15 (55.6%)			
Yes	8 (24.2%)	25 (75.8%)			
CTS5			–	–	–
< 3.13	12 (26.7%)	33 (73.3%)			
≥ 3.13	0 (0)	6 (100%)			

PR, progesterone receptor; CTS5, the Clinical Treatment Score post–5 years. The bold values provided for making meaningful result (p<0.05) stand out.

In univariate analysis of the previously-AI-treated group, age (OR 0.400, 95% CI 0.186-0.860, *p =* 0.019), pT (OR 2.844, 95% CI 1.483–5.454, *p =* 0.002), pN (OR 29.731, 95% CI 10.939–80.809, *p* < 0.001), HER-2 (OR 2.670, 95% CI 1.078–6.613, *p =* 0.034), Ki-67 status (OR 2.107, 95% CI 1.140–3.893, *p =* 0.017), Grade (GIII *vs.* GI: OR 7.778, 95% CI 2.032–29.773, *p =* 0.003), receipt of chemotherapy (OR 7.802, 95% CI 3.920–15.528, *p* < 0.001), and receipt of radiotherapy (OR 2.596, 95% CI 1.389–4.852, *p =* 0.003) were correlated with EET (**Table 5**). Multivariate analysis suggested that age (OR 0.315, 95% CI 0.117–0.848, *p =* 0.022), pN (OR 20.533, 95% CI 7.249–58.158, *p* < 0.001), and receipt of chemotherapy (OR = 4.387, 95% CI 1.893–10.169, *p =* 0.001) were independent predictors for the recommendation of EET (**Table 3**).

**Table 5:** Univariate analysis of impact factors for extended endocrine therapy recommendation in AI group.

AI(*n* = 175)	EET recommendation		OR	*p*	CI 95%
	No-EET	EET			
**Age, years**			**0.400**	**0.019**	**0.186–0.860**
**≤ 60**	**11 (25.6%)**	**32 (74.4%)**			
**> 60**	**61 (46.2%)**	**71 (53.8%)**			
Menopause status			0.404	0.181	0.107–1.525
Pre	3 (23.1%)	10 (76.9%)			
Post	69 (42.6%)	93 (57.4%)			
**pT**			**2.844**	**0.002**	**1.483–5.454**
**pT1**	**53 (51.0%)**	**51 (49.0%)**			
**pT2+**	**19 (26.8%)**	**52 (73.2%)**			
**pN**			**29.731**	**< 0.001**	**10.939–80.809**
**pN0**	**67 (67.7%)**	**32 (32.3%)**			
**PN1+**	**5 (6.6%)**	**71 (93.4%)**			
PR status			0.969	0.933	0.463–2.028
**Negative**	15 (40.5%)	22 (59.5%)			
**Positive**	57 (41.3%)	81 (58.7%)			
**HER-2 status**			**2.670**	**0.034**	**1.078–6.613**
**Negative**	**65 (44.8%)**	**80 (55.2%)**			
**Positive**	**7 (23.3%)**	**23 (76.7%)**			
**Ki67 status**			**2.107**	**0.017**	**1.140–3.893**
**< 15**	**44 (50.0%)**	**44 (50.0%)**			
**≥ 15**	**28 (32.2%)**	**59 (69.8%)**			
**Grade**				**0.008**	
**I**	**10 (62.5%)**	**6 (37.5%)**			
**II**	**40 (41.2%)**	**57 (58.8%)**	**2.375**	**0.120**	**0.799–7.063**
**III**	**6 (17.6%)**	**28 (82.4%)**	**7.778**	**0.003**	**2.032–29.773**
Operation methods			1.478	0.227	0.784–2.786
Lumpectomy	28 (47.5%)	31 (52.5%)			
Mastectomy	44 (37.9%)	72 (62.1%)			
**Chemotherapy**			**7.802**	**< 0.001**	**3.920–15.528**
**No**	**47 (70.1%)**	**20 (29.9%)**			
**Yes**	**25 (23.1%)**	**83 (76.9%)**			
**Target therapy**			2.488	0.066	0.941–6.582
**No**	66 (44.0%)	84 (56.0%)			
**Yes**	6 (24.0%)	19 (76.0%)			
**Radiotherapy**			**2.596**	**0.003**	**1.389–4.852**
**No**	**38 (55.1%)**	**31 (44.9%)**			
**Yes**	**34 (32.1%)**	**72 (69.7%)**			
**CTS5**			**34.375**	**0.001**	**4.550–259.724**
**< 3.13**	**55 (49.5%)**	**56 (50.5%)**			
**≥ 3.13**	**1 (2.8%)**	**35 (97.2%)**			

PR, progesterone receptor; CTS5, the Clinical Treatment Score post–5 years. The bold values provided for making meaningful result (p<0.05) stand out.

### The retrospective validation of CTS5 for EET decision making

In this study, 198 patients had data on CTS5. The distribution of CTS5 was 78.8% and 21.2% for the low (< 3.13) and high-risk (≥ 3.13) groups, respectively. Overall, CTS5 (OR 36.865, 95% CI 2.846–168.309, *p =* 0.001) was correlated with EET in univariate analysis (**Table 2**). After excluding the factors involved in the CTS5 formula, CTS5 (OR 21.887, 95% CI 2.846–168.309, *p =* 0.003) remained an independent predictor for the recommendation of EET in multivariate analysis (**Table 3**).

For patients previously SERM-treated, all patients were suggested to extend the endocrine therapy when their CTS5 status was indicated as high risk (**Table 4**).

For patients previously AI-treated, 35 (97.2%) patients were recommended EET in the previously AI-treated group when their CTS5 status was indicated as high risk. In the univariate analysis of the previously AI-treated group, CTS5 (OR 34.375, 95% CI 4.550–259.724, *p =* 0.001) was correlated with EET (**Table 5**). In multivariate analysis, CTS5 (OR 25.191, 95% CI 3.240–195.841, *p =* 0.002) remained an independent predictor for the recommendation of EET (**Table 3**).

### Impact of adverse events on decision making

In our study, the following common adverse events after endocrine therapy were recorded and analyzed: endometrial thickening, endometrial cancer, musculoskeletal symptoms, *T*-score < -2, fracture, hot flash (≥ G3), libido decreased (≥ G2), depression or anxiety, and dyslipidemia.

Of all patients who participated in the multidisciplinary discussion (*n* = 235), 14 patients had endometrial thickening (6%), one patient had endometrial cancer (0.4%), 19 patients had musculoskeletal symptoms (8.1%), 46 patients had osteoporosis (19.6%), 14 patients had fracture(6%), nine patients had hot flash (3.8%), six patients had libido decreased (≥ G2) (2.6%), 43 patients had depression or anxiety(18.3%), and 58 patients had dyslipidemia (24.7%) (**Table 6**). None of these AEs were significantly correlated with the recommendation of EET in univariate analysis. In patients previously treated with SERM or AI, similar results were found that none of these adverse events were correlated with treatment decisions (**Table 6**).

**Table 6:** The distribution of side effects in enrolled breast cancer patients and the correlation between AEs and extended endocrine therapy in whole patients enrolled and patients previously treated with SERM or AI.

	All patients enrolled (*N* = 235)			Post-treatment with SERM (*N* = 60)			Posttreatment with AI (*N* = 175)		
	No-EET	EET	*p*	No-EET	EET	*p*	No-EET	EET	*p*
Endometrial Thickening (*n* = 14, 6%)									
No	78 (40.2%)	116 (59.8%)	0.164	18 (36.7%)	31 (63.3%)	0.476	60 (41.4%)	85 (58.6%)	0.649
Yes	3 (21.4%)	11 (78.6%)		2 (22.2%)	7 (77.8%)		1 (20.0%)	4 (80.0%)	
Endometrial cancer (*n* = 1, 0.4%)									
No	91 (38.9%)	143 (61.1%)	0.374	20 (26.7%)	40 (73.3%)	–	72 (41.4%)	102 (58.6%)	1
Yes	1 (100%)	0 (0)		0 (0)	0 (0)		0 (0)	1 (100%)	
Musculoskeletal symptoms (> G2) (*n* = 19, 8.1%)									
No	87 (40.3%)	129 (59.7%)	0.114	20 (34.5%)	38 (65.5%)	0.548	64 (40.5%)	94 (59.5%)	0.602
Yes	5 (26.3%)	14 (73.7%)		0 (0)	2 (100%)		6 (75%)	2 (25%)	
T-score ≤ -2 ( (n = 46, 19.6%))									
No	79 (42.7%)	106 (57.3%)	0.059	19 (35.8%)	34 (64.2%)	0.161	52 (39.1%)	81 (60.9%)	0.344
Yes	11 (23.9%)	35 (76.1%)		0 (0)	5 (100%)		19 (47.5%)	21 (52.5%)	
Fracture (*n* = 14, 6%)									
No	86 (38.9%)	135 (61.1%)	0.769	25 (42.3%)	34 (57.6%)	/	68 (42.0%)	94 (58.0%)	0.430
Yes	6 (42.9%)	8 (57.1%)		0 (0)	1 (100%)		4 (30.8%)	9 (69.2%)	
Hot flash (≥ G3) (*n* = 9, 3.8%)									
No	89 (39.4%)	137 (60.6%)	0.503	19 (33.3%)	38 (66.7%)	1	70 (41.4%)	99 (58.6%)	1
Yes	3 (33.3%)	6 (66.7%)		1 (33.3%)	2 (66.7%)		2 (33.3%)	4 (66.7%)	
Libido decreased (≥ G2) (*n* = 6, 2.6%)									
No	89 (38.9%)	140 (61.1%)	0.681	18 (32.1%)	38 (67.9%)	0.595	72 (41.6%)	101 (58.4%)	0.513
Yes	3 (50%)	3 (50%)		2 (50.0%)	2 (50.0%)		0 (0)	2 (100%)	
Depression (*n* = 43, 18.3%) or anxiety									
No	73 (38.0%)	119 (62.0%)	0.454	16 (33.3%)	32 (66.7%)	1	60 (41.7%)	84 (58.3%)	0.762
Yes	19 (44.2%)	24 (55.8%)		4 (33.3%)	8 (66.7%)		12 (38.7%)	19 (61.3%)	
Dyslipidemia (*n* = 58, 24.7%)									
No	72 (40.7%)	105 (59.3%)	0.402	18 (34.6%)	34 (65.4%)	0.707	57 (45.6%)	68 (54.4%)	0.058
Yes	20 (34.5%)	38 (65.5%)		2 (25%)	6 (75%)		15 (30.0%)	35 (70.0%)	

## Discussion

Extending the duration of the endocrine therapy to 10 years has now proved to reduce the risk of late recurrence in selected ER+ breast cancer patients (4, 5, 10, 21). However, controversies remain about the target population who may benefit from the EET in clinical decision making. In our study, there were 235 ER-positive patients participated in the multidisciplinary discussion, and we found that age, lymph node status, and receipt of chemotherapy were independently associated with the recommendation of EET.

Among classic clinicopathological factors, nodal status is the strongest predictor of early recurrence (22). The study by HongChao and colleagues included 62,923 ER+ breast cancer patients and reported that the annual risk of distant recurrence was strongly related to nodal status (*P* < 0.001), and recurrence increased with the number of metastatic lymph nodes (20-year risk with N0, N1, and N2: 22%, 31%, and 52%) (5). 2018 ASCO guideline also recommended that women with node-positive breast cancer receive extended therapy, including AI, for up to a total of 10 years of adjuvant endocrine treatment (11). In our study, lymph node status turned out to be the strongest factor associated with therapy recommendation in all clinicopathological indicators. This indicated that clinicians would pay more attention to lymph node status when they make a recommendation on whether to use EET or not.

In our study, we found that age did not affect the recommendation for extended SERMs in the previously SERM-treated group, but it was the independent predictor for recommendation in the previously AI-treated group and older patients are less likely to be recommended for extended AIs.

At present, there is no evidence that age is related to the risk of recurrence (2). A meta-analysis in 2017 shows that there was no statistically significant benefit from extended therapy in the age subgroup (23). Therefore, for those who were previously treated with SERMs, age will not affect the choice of doctors. However, in patients previously treated with AI, the proportion of elderly patients (age > 60) is relatively large (75%) in our data. In the face of those patients, considering the physical condition, tolerance, and the lack of evidence to prove the validity of EET, clinicians tend to make the relatively conservative decision.

CTS5 (ATAC), including tumor size, number of positive nodes, histologic grade, and age, is a simple tool that was validated as highly prognostic for late recurrence (7, 24). In Dowsett’s research, the prognostic value of CTS5 was tested using data from the ATAC trial and validated with data from the BIG 1-98 trial (20). Furthermore, populations of those clinical trials are all postmenopausal patients and may behave differently to real-life patients. In their follow-up study, CTS5 demonstrated clinical validity for predicting late recurrence in unselected postmenopausal patients but less so in premenopausal patients (25). In our study, CTS5 was used for retrospective validation of the physician’s clinical recommendations about EET. Consistent with the experimental conclusion mentioned previously, we found that CTS5 was strongly associated with clinician recommendations, especially in the previously AI-treated group, and the higher the value, the more likely EET would be recommended. In addition, from the perspective of the OR values in the multivariate analysis, the CTS5 score was a more valuable guiding factor for the EET recommendation than lymph nodes and other independent clinicopathological factors.

Endocrine therapy causes some side effects, most of which were non-life threatening. In the IBIS-II trial, John et al. reported the side-effect profiles in breast cancer patients who had completed 5 years of endocrine therapy, including fractures (9%), arthralgia (57%), and osteoporosis (7%) with anastrozole and gynecological cancers (1.9%), vaginal symptoms (28%), and deep vein thromboses (1%) with tamoxifen (26). There are also some studies showing a possible side effect of tamoxifen with raise in the triglycerides level (27, 28). In our study, the side effects we counted were mainly *T*-score< -2 (26.7%), musculoskeletal symptoms (5.3%), and fracture (8.7%) with AI and endometrial thickening (15.5%) and dyslipidemia (13.3%) with tamoxifen.

EET would also increase the incidence of some side effects. In the NSABP B-14 trial, the risk of endometrial cancer was raised in the extended tamoxifen group [RR:2.0 (0.7–6.6)]. As reported by MA-17R, extended letrozole significantly increased the risk of osteoporosis (12%:9%, *p =* 0.01) and fracture (14%:9%, *p =* 0.001) (10, 29, 30). In our study, there was no influence of adverse events on the treatment decision. First, this might be related to the fact that the population enrolled in this study was able to tolerate 5 years of basic adjuvant endocrine therapy and was likely to endure further EET. Secondly, we consider that if complications would occur, priority would be given to the change of treatment or to treat complications aggressively rather than stopping EET.

There are some limitations to our study: one is that it was a retrospective analysis, which needs further validation. Next in importance, our data included cases from October 2017 to December 2019. Since then, the publication of clinical trial results and the update of clinical guidelines would lead to a change in decision making.

## Conclusions

Our study indicated that age, lymph nodal status, and receipt of chemotherapy were independent predictors for the recommendation of EET. The application of the CTS5 on EET decision making might be valuable among ER+ breast cancer patients.

## Data availability statement

The raw data supporting the conclusions of this article will be made available by the authors, without undue reservation.

## Author contributions

WLC: Conceptualization, Writing - Original Draft, Formal analysis, Statistical Analysis. JW: Methodology, Visualization. YZ, JHH: Investigation. XC: Data Curation. OH, JRH: Validation. YL, WGC, KS: Project administration, Supervision. LZ: Writing- Reviewing and Editing. All authors contributed to the article and approved the submitted version.
